# Is it feasible to use smartphone images to perform telediagnosis of different stages of occlusal caries lesions?

**DOI:** 10.1371/journal.pone.0202116

**Published:** 2018-09-06

**Authors:** Eduardo K. Kohara, Camilla G. Abdala, Tatiane F. Novaes, Mariana M. Braga, Ana E. Haddad, Fausto M. Mendes

**Affiliations:** 1 Department of Pediatric Dentistry, School of Dentistry, University of São Paulo, São Paulo, SP, Brazil; 2 School of Dentistry, University of Guarulhos, Guarulhos, SP, Brazil; 3 School of Dentistry, Cruzeiro do Sul University, São Paulo, SP, Brazil; 4 Teledentistry and Telehealth Center, School of Dentistry, University of São Paulo, São Paulo, SP, Brazil; University of Queensland, AUSTRALIA

## Abstract

The purpose of this study was to compare the performance of two different models of smartphone and a conventional camera with that of direct clinical examination in detecting caries lesions at different stages of progression in deciduous molars. The photographic equipment consisted of two smartphones (iPhone and Nexus 4) and a conventional macro camera setup. First, in the laboratory phase of the study, we compared the images of 20 exfoliated primary teeth having caries lesions at different stages. Then, in the clinical phase of the study, the images of 119 primary molars from fifteen children (3 to 6 years old) were used. All of the photographic images were taken using the previously described devices. In both groups, two examiners, blinded to the photographic equipment used, assessed the images independently on a computer screen, and classified them according to the International Caries Detection and Assessment System (ICDAS). The teeth were then examined directly by two other experienced examiners, and the consensus reached was considered the reference standard. Parameters of validity, such as percentage of correct answers, agreement with the reference standard, sensitivity, specificity and inter-examiner agreement (using the weighted kappa test) were calculated. The examiners performed similarly in both *in vitro* and *in vivo* studies. Inter-examiner reliability was approximately 0.7 for all the devices in the laboratory setting, and for the macro camera photography system in the clinical setting, but it was approximately 0.9 for the iPhone and Nexus images taken *in vivo*. With regard to the percentage of correct answers, the highest values were observed for sound and extensive caries lesions in both laboratory and clinical settings. The percentage of correct answers for initial and moderate lesions was particularly low in the clinical evaluation, irrespective of the camera devices used. Therefore, we concluded that photographic diagnosis using smartphone images is feasible and accurate for distinguishing sound tooth surfaces from extensive caries lesions; however, photographic images are not a good method for accurately detecting initial and moderate caries lesions.

## Introduction

Dental caries is the most prevalent chronic disease worldwide [1]. This disease causes localized destruction of dental tissues, known as caries lesions or dental decay, which is the result of mineral loss from dental tissues. Acids resulting from the metabolism of carbohydrates by bacteria present in the oral environment cause this mineral loss. Although dental cavities are usually recognized as the definitive signs of dental caries, these are merely the later, irreversible stages of the disease. At earlier stages, caries lesions are characterized by a change in the appearance of the tooth surface, from shiny and clear, in sound enamel, to rough, whitish and opaque. This characteristic is the result of the loss of mineral content from enamel surfaces and subsurfaces, and is so distinct that lesions at this stage are also called white spots.

Currently, the diagnostic process of dental decay comprises the detection of both early (initial lesions or white spots) and advanced (cavitated) stages of dental caries. The most widely studied caries diagnosis system is the International Caries Detections and Assessment System (ICDAS) [2], and there are a number of articles reporting only visual examination without additional methods as the best caries diagnosis strategy [3–7]. This would make visual examination the first choice for caries lesions detection.

Nevertheless, conventional visual inspection does not provide a physical record of the teeth examined, in comparison with other methods, such as dental radiography. Using a method for remote discussion, such as photography, could bring about a substantial improvement in dental education and case discussion, since it would enable discussing clinical situations at a distance by sharing exam results with professionals and/or students based at different locations. Health Services are gradually adopting this concept in Telemedicine and Teledentistry initiatives, with encouraging results, especially in educational and diagnostic applications [8]. This could, however, be more challenging in the field of cariology, in that early caries lesions are clinically distinguished from the adjacent sound tissues by differences in shades of white. Using an improper technique or low-quality equipment would lead to low-quality images, and consequent incorrect diagnosis and questionable treatment decision.

Dental photography is a very technique-sensitive method, owing to the closed, humid and dark environment in the mouth, and to the interaction between light and dental tissues. The current recommendation is to use an interchangeable-lens camera (also known as single reflex camera) coupled to a macro lens with 1:1 magnification to record images with optimal quality, with a macro-twin or ring-flash as light source [9]. The disadvantages of this system are size and cost. In contrast, smartphones equipped with cameras are very popular and relatively inexpensive. Furthermore, their portability enables dental professionals to carry their own personal equipment, record images of interest at any location, and easily send them to colleagues or instructors for discussion. Nevertheless, the image quality provided by these cameras remains a challenge [10].

Some authors have published articles reporting on the photographic diagnosis of caries lesions [11–13]. However, we found no reports investigating the feasibility of using a smartphone camera in diagnosing dental caries throughout all stages of the disease. Therefore, the aim of this study was to assess the performance of visual caries detection in the occlusal surfaces of primary teeth, using images recorded by smartphones equipped with cameras and by a dedicated macro photography system, compared with that of direct clinical examination.

## Material and methods

### Study design and ethical considerations

This study on the accuracy of visual inspection performed using photographic images for detecting occlusal caries lesions in primary molars was divided into two phases, namely *in vitro* and *in vivo*. The *in vitro* stage aimed to establish standard photographic settings and diagnostic training for the *in vivo* phase. We decided to use only occlusal surfaces because diagnosing these surfaces is more challenging than diagnosing smooth surfaces. Moreover, it would be unfeasible to detect caries lesions using images of proximal surfaces. This study was approved by the Research Ethics Committee, School of Dentistry, University of São Paulo, on June 29, 2012 (register number 02952612.4.0000.0075).

### Photographic equipment

All photographic images used in this study were recorded using three different camera systems, with different standardized settings. Initially, we conducted a laboratory study to adjust the settings of all the photographic systems used in the study. After establishing optimal settings, we carried out the *in vitro* experiment. We then conducted the clinical phase of the study using these same settings. Further minor adjustments were required in this phase.

The following smartphones were used: Apple iPhone 5 (Apple Inc, Cupertino, CA, USA) and LG Google Nexus 4 (LG Electronics, Seoul, South Korea). Both had fully automatic settings for image capture, and image size was set at 2448 x 3264 pixels (8 megapixels CCD sensor).

The macro photographic system consisted of a Panasonic DMC-G2 body (Matsushita Electric Industrial Co., Ltd., Osaka, Japan) coupled to an Olympus M. Zuiko Macro Digital ED 60 mm 1:2.8 lens (Olympus Corp., Tokyo, Japan) and a ring-light flash (Sunpak D-Macro, Sunpak Co., Ltd., Tokyo, Japan). Settings were defined as follows: “M” mode, 1/160 s exposure time, f/22 aperture, “flash” white balance, ISO 200, and flash power set to 1/8. The size of the images produced with this camera was 4000 x 3000 pixels (12 megapixels CCD sensor).

### In vitro experiment

#### Dental specimens

For this part of the study, we used 11 first molars and 9 second molars, obtained from The Human Teeth Bank of the School of Dentistry, University of Sao Paulo. These teeth were naturally exfoliated or were extracted for orthodontic reasons. They presented occlusal surfaces with different stages of tooth decay, varying from sound enamel to extensive carious lesions. In second primary molars, two sites were chosen and assessed separately. Thus, the final sample comprised 29 sites in 20 teeth. We used the ICDAS system to classify the surfaces in the photographic images. The description of ICDAS scores can be found elsewhere [14]. However, to simplify the assessment, we used the merged scores of the ICDAS, as proposed previously [15]. Therefore, the surfaces rated as scores 1 and 2 according to the original ICDAS criteria were classified as initial caries lesions, those rated as scores 3 and 4 were classified as moderate caries lesions, and those rated as scores 5 and 6 were classified as extensive caries lesions. Moreover, we did not evaluate the activity status of the lesions in the present study.

#### Image capture and analysis

The same operator (EKK) took all the photographic images. Tooth images were recorded in the same room, one at a time, using the three pieces of equipment sequentially to avoid changes in lighting conditions. Room illumination consisted of four 40 W white fluorescent bulbs (Philips, Brazil). The distance between tooth and camera was standardized at 30 cm, and the teeth were positioned with the caries lesions facing upward to facilitate photograph taking. When photographing with smartphones, we selected the metering point by tapping the tooth image on the screen and touching the corresponding command, before taking the picture.

One week after the photographic images were recorded, they were randomly transferred to two computers, with no record of the photographic system used for each picture. Then, two examiners (EKK and CGA), blinded to the photographic systems used, analyzed the images independently and attributed an ICDAS score to each lesion. The brightness and contrast levels of the computer screens were set by each examiner, based on their individual preference, and then kept until the end of the analysis, performed with Windows Photo Viewer software (Microsoft Inc., Redmond, Washington, USA). No post-production adjustment was made to the images, with the exception of a zoom-in until the tooth image was sufficiently enlarged on the computer screen to make the diagnostic process possible.

#### Reference standard and statistical analysis

As a reference standard, two benchmark examiners (TFN and MMB), experienced in ICDAS scoring and unware of the results of previous examinations, evaluated all the dental surfaces by direct visual inspection and attributed a score to each tooth. The examinations were made independently by the examiners. The teeth were first examined wet, then were dried for 5 s, and examined again under the illumination provided by a dental unit light. In case of discrepancy between examiners, they conferred until reaching a consensus, and this consensual score was considered as the reference standard.

Weighted kappa values and respective 95% confidence intervals (95% CI) were calculated to assess the agreement between the evaluations made using the photographic images obtained with the different devices and the reference standard. Comparisons between the devices were made by analyzing the 95% CI values. We also calculated the percentage of right answers considering sound enamel surfaces and the different stages of the caries process. We performed this analysis by grouping the original ICDAS scores into the categories of initial, moderate and extensive lesions [15]. Non-cavitated lesions (ICDAS scores 1 and 2) were classified as initial, lesions rated as ICDAS score 3 or 4 were classified as moderate, and lesions with dentin cavitation (ICDAS scores 5 and 6) were classified as extensive. These analyses were made separately by each examiner. We also calculated the inter-examiner reproducibility using the weighted kappa test. After concluding the data analysis for the *in vitro* study, we went on to conduct the clinical study.

### In vivo study

#### Participants

Fifteen children aged 3 to 6 years participated in this study. The parents or legal guardians of the children sought dental treatment for them at the Clinic of the Department of Pediatric Dentistry, School of Dentistry, University of São Paulo. All the participants were enrolled in a broader clinical trial on caries diagnostic strategies for primary teeth (Caries Detection in Children–CARDEC trials, registered at the Clinical Trials platform under register number NCT02078453). After agreeing to participate and having consent forms signed by their legal caretaker, each child was consecutively taken to a dental unit for image collection and clinical examination. Inclusion criteria were children with good behavior, good health conditions, and the presence of one or more primary molars. We excluded from the study children whose parents or guardians did not agree to participate in this study, and children who presented non-cooperative behavior during the examination and image recording sessions.

We did not calculate the sample size previously, because the present study was nested in a larger clinical trial; therefore, we decided to use a convenience sample. Nonetheless, after applying the McNemar test and considering a type I error of 5%, we established that our sample needed a power of 0.82 to detect a difference of 20% in the study parameters.

#### Image collection and lesion detection

The same operator (EKK) recorded all the images of the occlusal surfaces of primary molars. The illumination provided by the dental unit light was used during the image recording sessions carried out with both smartphones. The dental unit light was turned off when taking photographs with the macro camera system, during which the only light source used was the ring flash.

Before taking the pictures, dental prophylaxis cleaning was performed with prophylactic paste, rubber cups and rotating brushes powered by a low-speed turbine. Pictures were taken after reflecting the tooth images on a mouth-sized photographic metal mirror (Ref. 13573, YDM Corporation, Japan). Air from the dental unit syringe was blown onto the mirror surface to prevent moisture buildup. The teeth were also dried, and a saliva ejector and cotton rolls were kept in the patient’s mouth during the procedure, when necessary. Just as in the *in vitro* study, the metering point was set by tapping the tooth image on the screen of the smartphone cameras before touching the image acquisition button. We sequentially produced images of all quadrants with each camera at a time. The images used in this study showed teeth and oral tissues only; we did not record images that would permit facial recognition.

Forty-five days after the images were recorded, they were randomly transferred to two computers, irrespective of the photographic equipment used. The images of the occlusal surfaces of primary molars were then assessed independently in the same way as in the laboratory experiment. We chose one site per occlusal surface in this assessment, and did not select teeth with dental restorations. The same examiners (EKK and CGA) attributed an ICDAS score to each occlusal surface. The sole adjustment made during image assessment was a zoom-in until the tooth image was sufficiently large to make lesion detection possible, using Windows Photo Viewer software. The examiners were unaware of each other’s results or of the clinical condition of the dental surfaces.

#### Reference standard and statistical analysis

The children had their teeth examined sequentially by two experienced clinicians (TFN and MMB). The examinations were conducted by direct visual inspection, with the children positioned in a dental chair. The examiners used plane mouth mirrors and a ball-ended probe, and the teeth were assessed under illumination, after they had been cleaned with a rotating bristle brush and pumice/water slurry. The benchmark examiners evaluated the teeth independently, and, in case of discrepancy, they conferred until reaching a consensus. The score attributed by the benchmark examiners was considered the correct answer for each tooth surface.

In the statistical analysis, inter-examiner reproducibility was calculated using the weighted kappa test. With regard to accuracy, concordance between the assessments made based on the photographic images, and the reference standard was also calculated by the weighted kappa test. In addition, the percentage of correct answers was calculated separately for sound surfaces, and for initial, moderate and extensive lesions. In this part of the study, we also calculated sensitivity, specificity and the respective 95% CIs for the assessments made using the pictures, considering three different thresholds: initial (sound surfaces vs initial, moderate and extensive lesions), moderate (sound surfaces and initial lesions were considered as a sound surface, whereas moderate and extensive lesions were considered as decayed), and extensive caries lesions (surfaces with no lesions, with initial or moderate lesions were classified as sound surfaces, whereas surfaces with extensive caries lesions were considered as having the disease). Again, the assessments were made separately by each examiner.

Statistical differences among the values were considered when there was no interpolation of 95%CI values. All the analyses were made using an appropriate statistical software (MedCalc 13.3.3.0, Mariakerke, Belgium).

## Results

In the laboratory phase of the study, the occlusal surfaces were classified by the two experienced examiners as follows: 2 sites were classified as sound (6.9%), 9 had initial caries lesions (31.0%), 10 sites had moderate lesions (34.5%) and 8 sites had extensive caries lesions (27.6%) (Table 1). The diagnoses made according to the images recorded by the three different camera devices were similar to those made during the direct clinical examination of the dental surfaces. The kappa values obtained when testing agreement with the reference standard ranged from 0.559 to 0.733. The percentage of agreement was higher for sound surfaces and extensive caries lesions than for initial and moderate caries lesions (Table 1).

**Table 1:** Agreement between assessments and number (and %) of correct assessments made by two examiners during the *in vitro* detection of caries lesions in pictures of tooth surfaces taken with different camera devices (a macro camera and two smartphones), using the International Caries Detection and Assessment System (ICDAS), as compared with direct visual inspection.

Camera devices	Weighted kappa values (95% CI)	Sound surfaces	Initial lesions	Moderate lesions	Cavitated lesions
		N (%)			
Direct clinical assessment		2	9	10	8
**Examiner 1**					
Macro	0.617 (0.360 to 0.874)	2 (100.0)	5 (55.6)	4 (40.0)	6 (75.0)
iPhone	0.559 (0.318 to 0.800)	2 (100.0)	4 (44.4)	2 (20.0)	6 (75.0)
Nexus	0.541 (0.301 to 0.781)	2 (100.0)	4 (44.4)	1 (10.0)	6 (75.0)
**Examiner 2**					
Macro	0.650 (0.414 to 0.886)	2 (100.0)	6 (66.7)	4 (40.0)	7 (87.5)
iPhone	0.733 (0.534 to 0.932)	2 (100.0)	3 (33.3)	9 (90.0)	7 (87.5)
Nexus	0.619 (0.388 to 0.851)	2 (100.0)	3 (33.3)	5 (50.0)	7 (87.5)

95% CI = 95% confidence intervals

Table 2 shows the weighted kappa values for the inter-examiner reproducibility between the two examiners, using images obtained in laboratory and clinical settings. Values ranged from 0.605 to 0.768 in the *in vitro* experiment. The mobile devices presented higher kappa inter-examiner reliability values than the macro camera in the clinical setting (Table 2). The inter-examiner reliability between the two benchmark examiners who performed the direct clinical assessment of the teeth (reference standard) attained weighted kappa values higher than 0.80 in both laboratory and clinical settings.

**Table 2:** *In vitro* and *in vivo* inter-examiner reliability between the two examiners in detecting caries lesions in pictures of tooth surfaces taken with different camera devices, using the International Caries Detection and Assessment System (ICDAS).

Camera devices	Weighted kappa values (95% confidence interval)	
	*In vitro*	*In vivo*
Macro	0.768 (0.582 to 0.954)	0.695 (0.549 to 0.842)
iPhone	0.615 (0.382 to 0.847)	0.973 (0.962 to 0.985)
Nexus	0.605 (0.357 to 0.853)	0.895 (0.796 to 0.994)

In the clinical part of the experiment, and after reaching a consensus, the benchmark examiners classified 6 (5.0%) occlusal surfaces as sound, 64 (53.8%) as having initial caries lesions, 29 as having moderate caries lesions (24.4%), and 20 (16.8%) as having extensive caries lesions. Low weighted kappa values (lower than 0.66) were obtained for all devices when testing the agreement among the assessments made on the images obtained in the clinical setting and the reference standard. Moreover, similar diagnostic performance was observed for the images taken with the three devices. The agreement percentages were much higher for sound surfaces and extensive caries lesions (higher than 75%). In contrast, detection of initial and moderate caries lesions was very poor for all camera devices. In some situations, the percentage of correct answers was lower than 10% for these subgroups (Table 3).

**Table 3:** Agreement between assessments and number (and %) of correct assessments made by two examiners during the *in vivo* detection of caries lesions in pictures of tooth surfaces taken with different camera devices (a macro camera and two smartphones), using the International Caries Detection and Assessment System (ICDAS), as compared with the consensual assessments made by two examiners performing direct visual inspection.

Camera devices	Weighted kappa values (95% CI)	Sound surfaces	Initial lesions	Moderate lesions	Cavitated lesions
		N (%)			
Direct clinical assessment		6	64	29	20
**Examiner 1**					
Macro	0.663 (0.566 to 0.760)	6 (100.0)	7 (10.9)	4 (13.8)	20 (100.0)
iPhone	0.638 (0.532 to 0.743)	6 (100.0)	3 (4.7)	2 (6.9)	19 (95.0)
Nexus	0.606 (0.498 to 0.714)	6 (100.0)	2 (3.1)	0 (0.0)	18 (90.0)
**Examiner 2**					
Macro	0.540 (0.404 to 0.677)	5 (83.3)	3 (4.7)	2 (6.9)	16 (80.0)
iPhone	0.636 (0.531 to 0.740)	6 (100.0)	11 (17.2)	2 (6.9)	17 (85.0)
Nexus	0.530 (0.403 to 0.656)	6 (100.0)	2 (3.1)	3 (10.3)	15 (75.0)

95% CI = 95% confidence intervals

This poor performance was also observed in terms of sensitivity, considering the initial and moderate caries lesion thresholds. The sensitivity values obtained in the detection of initial caries were lower than 0.40 for all the devices, and were also low considering moderate lesions as the threshold. On the other hand, when considering the detection of extensive caries lesions, the sensitivity values ranged from 0.750 to 1.000. There were no differences among devices and examiners considering all the thresholds (Table 4). Values of specificity were higher than 83%, and no differences were observed, irrespective of the examiner, camera or lesion threshold (Table 4). Examples of clinical images with an extensive carious lesion (S1 Fig) and with an initial caries lesion (S2 Fig) taken with the different photographic devices can be observed in the supporting information.

**Table 4:** Sensitivity and specificity of the assessments made by two examiners during the *in vivo* detection of caries lesions in pictures taken with different camera devices, using the International Caries Detection and Assessment System (ICDAS), and considering three different thresholds: initial lesions, moderate lesions and extensive lesions.

Camera devices	Sensitivity			Specificity		
	Initial	Moderate	Extensive	Initial	Moderate	Extensive
**Examiner 1**						
**Macro**	40.7 (31.6 to 50.4)	59.2 (44.2 to 73.0)	100.0 (83.2 to 100.0)	100.0 (54.1 to 100.0)	95.7 (88.0 to 99.1)	93.9 (87.3 to 97.7)
**iPhone**	33.6 (25.0 to 43.1)	53.1 (38.3 to 67.5)	95.0 (75.1 to 99.9)	100.0 (54.1 to 100.0)	98.6 (92.3 to 100.0)	93.9 (87.3 to 97.7)
**Nexus**	30.1 (21.8 to 39.4)	51.0 (36.3 to 65.6)	90.0 (68.3 to 98.8)	83.3 (35.9 to 99.6)	97.1 (90.1 to 99.7)	91.9 (84.7 to 96.4)
**Examiner 2**						
**Macro**	32.7 (24.2 to 42.2)	51.0 (36.3 to 65.6)	80.0 (56.3 to 94.3)	83.3 (35.9 to 99.6)	95.7 (88.0 to 99.1)	90.9 (83.4 to 95.8)
**iPhone**	39.8 (30.7 to 49.5)	53.1 (38.3 to 67.5)	85.0 (62.1 to 96.8)	100.0 (54.1 to 100.0)	98.6 (92.3 to 100.0)	95.0 (88.6 to 98.3)
**Nexus**	29.2 (21.0 to 38.5)	46.9 (32.5 to 61.7)	75.0 (50.9 to 91.3)	100.0 (54.1 to 100.0)	97.1 (90.1 to 99.7)	96.0 (90.0 to 98.9)

The figures in parentheses represent the 95% confidence intervals

## Discussion

The use of information and communication technologies (ICT) is growing in all fields, and especially in the field of healthcare, with telehealth and, in the case of this study, with teledentistry initiatives. The Brazilian Telehealth Program has been in place since 2007, with the partnership of municipalities and universities, and has provided teleconsulting for physicians, nurses, dentists and other health professionals working in the Unified Health System (SUS) [16, 17]. Today, the Brazilian Telehealth Program is available in 24 of the country’s 27 states, and in more than 2,000 cities, involving 30,000 health professionals from the Family Health Program. From 2008 to August 2016, it provided over 3 million telediagnoses, participated in 2.9 million tele-education activities, and delivered almost 500,000 teleconsultations and 1,039 second opinions [18]. A recent study conducted on three telemedicine services operating for more than ten years has found that an important factor of the services that last over time is whether or not they pursue a strategy that focuses on a specific need [19].

As far as telediagnosis is concerned, one of the requirements for success is the availability of good quality images. Therefore, studies are warranted on the image acquisition hardware requirements to provide reliable images for use in telediagnosis as a second opinion. This method has already been used in other fields of healthcare, with encouraging results in dermatology [20], otolaryngology [21] and ophthalmology [22], where smartphones with few or no adaptations were used. In the present study, we aimed to determine if similar results could be achieved in caries detection.

Previous studies have investigated the use of photographic images as a tool for caries lesion detection. A previous study compared the treatment decisions made based on intraoral images taken with a conventional camera capable of providing an image quality comparable to that provided by the smartphones used in our study [11]. In other studies [12, 23, 24], intraoral cameras were used, which are very convenient in clinical practice, but are not as portable as smartphones.

An earlier study has evaluated the performance of telediagnosis in caries detection based on images produced by a macro photography setup comprising an interchangeable lens camera coupled to a macro lens and a ring flash[25]. The authors found a significant association between direct clinical assessments and those performed using the images [25]. However, all of these studies considered only extensive and already cavitated caries lesions, and used images to calculate decayed, missing and filled deciduous teeth (dmf-t), as well as decayed, missing and filled permanent teeth (DMF-T). In general, they found the caries assessment made on the images to be feasible and similar to those performed clinically. In the present study, examinations made using smartphone and macro camera images were also able to differentiate sound from extensive caries lesions, thus corroborating their results.

We also found two studies in which the authors detected different ICDAS scores on photographic images. Unfortunately, the results of one of them are not comparable to ours, since this study aimed exclusively at comparing the caries lesion detection performance of newly developed software to that of an ICDAS-experienced dentist using the same photographic images [26]. No comparison with clinical examination data was reported in this previously published article [26]. The authors of the other study used images of the buccal aspect of teeth from patients that had recently completed orthodontic treatment, with caries lesions in different stages of progression [13]. They used a camera similar to our macro setup, consisting of a camera body coupled to a macro lens and a ring flash, and compared the images to the results of a clinical examination. Their agreement results for severe lesions were similar to ours, between 94.4% and 100% (ICDAS 5 and 6, respectively). Another important point is that caries lesions on buccal surfaces are technically easier to record than on occlusal surfaces, meaning that the examiner had better images to work with than those used in our study. In addition, the diagnosis of occlusal dental decay is more problematic than that of decay on the smooth surfaces of the tooth’s buccal aspect.

This good performance, however, was not detected when non-evident initial and moderate caries lesions were considered in the clinical setting. This difficulty was also observed in our study, since many lesions at these stages were not diagnosed as lesions. In fact, the performance of photographic images—even those taken with a macro camera—in enabling the detection of initial and moderate caries lesions were worse than that of direct visual inspection, in both the laboratory and clinical phases of our study [27]. This result was also observed in a previous study [13]. Although inter-examiner reproducibility had been high in the assessments made with smartphone images (mainly with the iPhone), this finding can be attributed to the high consistency observed between examiners in the errors made when detecting initial and moderate lesions, demonstrating that good agreement does not necessarily represent good performance.

These discrepancies between the detection made clinically and that made with images could be explained by the different shades of white of enamel lesions. With regard to the photographic technique, there are also difficulties in recording images of white spot lesions when the camera metering function automatically averages the entire image or the spot indicated on the display. Considering that teeth are the lightest objects in an intraoral image, it is not uncommon for them to be shown excessively bright, particularly if we fail to correct exposure values or to use a manual mode where the correct settings can be made based on the photographer’s own experience [28].

There is also another dental condition, namely fluorosis, whose clinical diagnosis is also based on the differences in shades of white on the tooth surface. Earlier studies have tested the use of photographic images to diagnosis this developmental enamel defect, and have reported that the diagnosis of fluorosis made on photographic images is as reliable as the clinical diagnosis [29, 30]. On the other hand, in these studies, the use of images of maxillary incisors, whose location provides better conditions for image acquisition than other dental groups, was a factor that certainly facilitated the photographic recording and subsequent diagnosis. In our study, the recording of occlusal images of molars in young children was a laborious process requiring different accessories and techniques, ultimately restricting the procedure of recording photographic images of enamel caries lesions. This poor performance was also observed when recording the images of exfoliated teeth; hence, another difficulty in achieving good-quality images could be related to the complex anatomy of primary molars, as well as to their location in the posterior part of the mouth.

Equipment selection in this study was based on the fact that the two devices chosen use the most popular operational systems for smartphones. Smartphones with similar characteristics of image output were used to minimize the influence of the file size when zooming in on the tooth image. It would be practically impossible to undertake a comparison of all the smartphones with built-in cameras available in the market, or of the most up-to-date ones, considering that companies launch new models with new functions and parts at a pace that is difficult to keep up with. An Android App has been created to facilitate the acquisition ans transmission of dental images to a store-and-forward based telemedicine server [31]. A recent systematic review on the use of teledentistry for caries detection conclude that although valuable, further studies, based on carefully designed research are still needed to investigate its effectiveness [32]. Thus, further studies could focus on the technique itself, as well as on testing other functions and settings, rather than testing a different range of smartphone hardware. Another possibility would be to use an intraoral camera in the comparison, the lack of which could be considered a limitation of the present study (this equipment was not available to us at the time the experiments were conducted). Poor performance in accurately detecting the earliest stages of the caries process in posterior teeth were observed, even when the macro camera setup was used, indicating that telediagnosis would not be a good indication for early lesions. Further research is warranted to evaluate the use of an intraoral camera for this purpose.

Another limitation of the present study is that all the photographic images were taken by the same operator, considering that the practice of telediagnosis implies that images should be taken by different clinicians at different locations. We decided to standardize this procedure to increase the internal validity of our study. Nevertheless, an interesting next step would be to test the feasibility of performing telediagnosis of caries lesions in a true clinical setting.

## Conclusions

We concluded that performing diagnoses based on photographic images provided by cameras is feasible, and that the diagnosis is accurate in distinguishing sound enamel surfaces from extensive (cavitated) lesions; however, this method is not accurate in detecting initial and moderate caries lesions. Both the smartphone cameras and the conventional macro cameras displayed similar performance.

## Supporting information

S1 Fig Representative images of an extensive lesion assessed in clinical study. OR shows a picture as recorded. Each image taken with an iPhone (iP), Nexus 4 (N4) and macro camera setup (MA) was then zoomed in on to allow photographic detection of lesions on a computer screen. In this image, the lesion detected in a primary maxillary second molar was assessed as being cavitated by all the photographic and reference examiners.
(JPG)

S2 Fig Representative zoomed-in images of an initial lesion assessed in the clinical study. Images were recorded using an iPhone (iP), a Nexus 4 (N4) and a macro camera setup (MA) to allow photographic detection of lesions on a computer screen. In this image, a primary mandibular first molar was classified as having an initial caries lesion by the benchmark examiners, but no lesion was detected by the photographic examiners.
(JPG)
