# Supplementary figures and images for: Is it feasible to use smartphone images to perform telediagnosis of different stages of occlusal caries lesions?
Source: PLoS One. 2018 Sep 6;13(9):e0202116. doi: 10.1371/journal.pone.0202116 (PMC6126822; doi:10.1371/journal.pone.0202116)

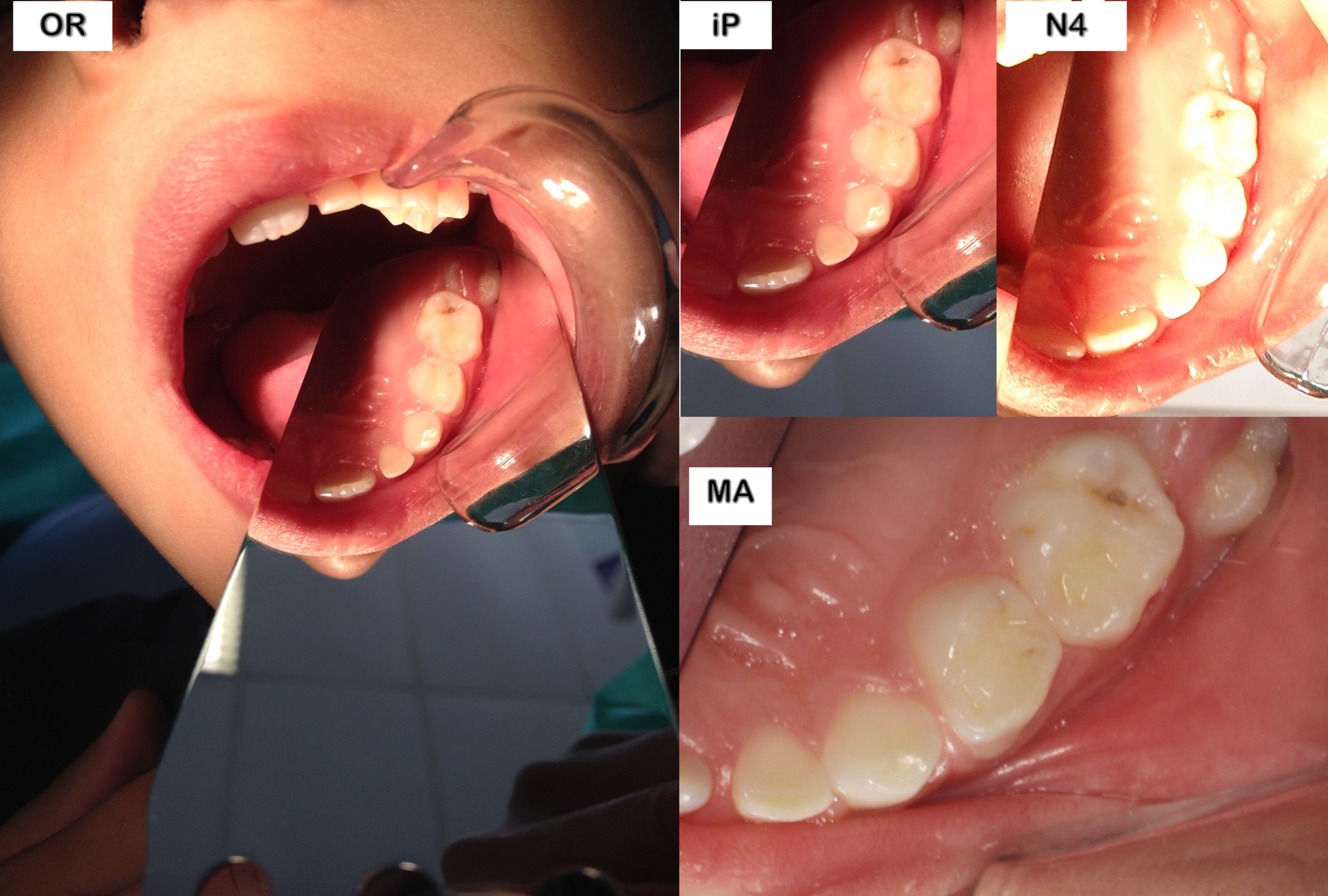

Supplement: S1 Fig — OR shows a picture as recorded. Each image taken with an iPhone (iP), Nexus 4 (N4) and macro camera setup (MA) was then zoomed in on to allow photographic detection of lesions on a computer screen. In this image, the lesion detected in a primary maxillary second molar was assessed as being cavitated by all the photographic and reference examiners. (JPG) [file pone.0202116.s001.jpg]

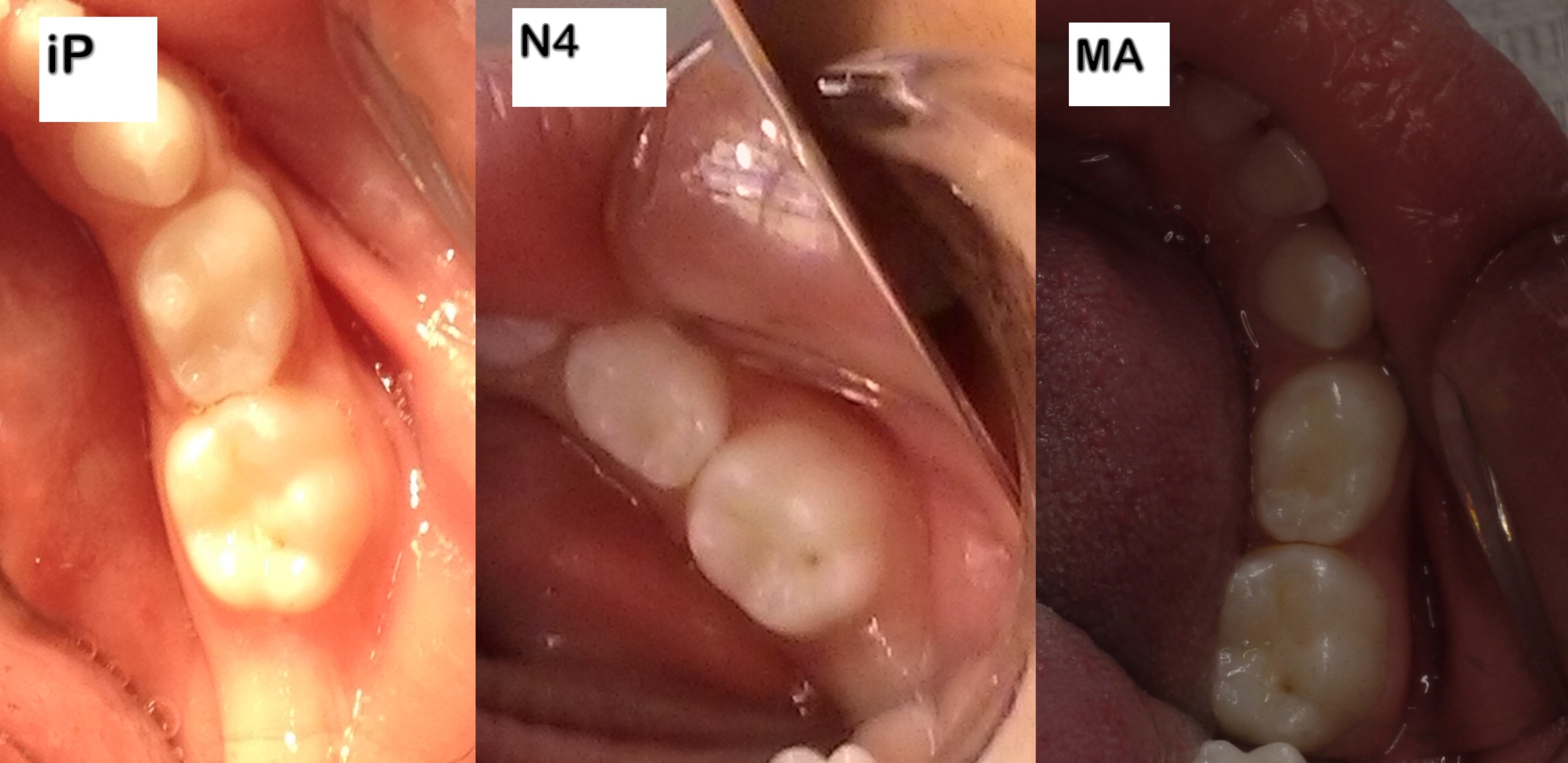

Supplement: S2 Fig — Images were recorded using an iPhone (iP), a Nexus 4 (N4) and a macro camera setup (MA) to allow photographic detection of lesions on a computer screen. In this image, a primary mandibular first molar was classified as having an initial caries lesion by the benchmark examiners, but no lesion was detected by the photographic examiners. (JPG) [file pone.0202116.s002.jpg]
